# Dressing Apraxia as Initial Manifestation of Creutzfeldt-Jakob Disease

**DOI:** 10.5334/tohm.72

**Published:** 2020-07-07

**Authors:** Josef G. Heckmann, Ivana Vachalova, Irina Vynogradova, Stefan Schwab

**Affiliations:** 1 Department of Neurology, Municipal Hospital Landshut, DE; 2 Department of Neurology, University Hospital Erlangen, DE

**Keywords:** Creutzfeldt-Jabob disease, dressing apraxia, neuropsychological disorder, movement disorder

## Abstract

**Background:**

Creutzfeldt-Jakob disease (CJD) is a rare prion disease characterized by rapidly progressive dementia.

**Case Report:**

A 76-year-old woman exhibited pronounced signs and symptoms of dressing apraxia for about seven weeks before the disease progressed and probable CJD was diagnosed supported by imaging and CSF findings.

**Discussion:**

Dressing apraxia as the initial manifestation of CJD has been sparsely reported. This remarkably focal syndrome should be considered with view on movement and neuropsychological disorders in early CJD.

## Introduction

Creutzfeldt-Jakob disease (CJD) is a rare prion disease mainly characterized by rapidly progressive dementia. Neuropsychological disorders however frequently occur in the initial phase of the disease with amnesia, impaired attention and frontal lobe syndrome as the most frequent [1]. Recently “arm levitation” as initial manifestation of Creutzfeldt-Jakob disease had been presented and judged as a manifestation of the alien limb phenomenon, which had been reported in the literature in 22 cases of CJD and was the first and exclusive manifestation in five of these cases [2]. By presenting a case report of dressing apraxia as the initial manifestation of CJD, we wish to emphasize the diversity of movement and neuropsychological disorders in CJD, and that initial symptoms can be very remarkably focal.

## Case Report

A 76-year-old woman developed difficulties in dressing. She had progressive problems dressing herself. In particular, she had difficulty handling buttons and arranging her clothing articles on her body. Dressing became more and more prolonged, and she began to manipulate her clothes incoherently until finally needing support from her husband. Her medical history included stable plasmocytoma, atrial fibrillation and residual effects from a minor stroke, and she was orally anticoagulated with phenprocoumon. Her neurological examination on admission revealed a slight right-sided motor impairment with finger tapping and slight dysarthria, which resulted from the previous minor stroke. Neuropsychologically pronounced signs and symptoms of dressing apraxia were found whereby performing simple gestures and pantomiming object use were preserved. Otherwise, there were no signs of constructional and limb ataxia, optic ataxia and visual disturbances. The MoCA test showed 28 of 30 points with slight impairment in the visuospatial items. Her family history was unremarkable. She had not had any previous neurosurgical procedures or corneal transplants [3].

**Figure 1:**
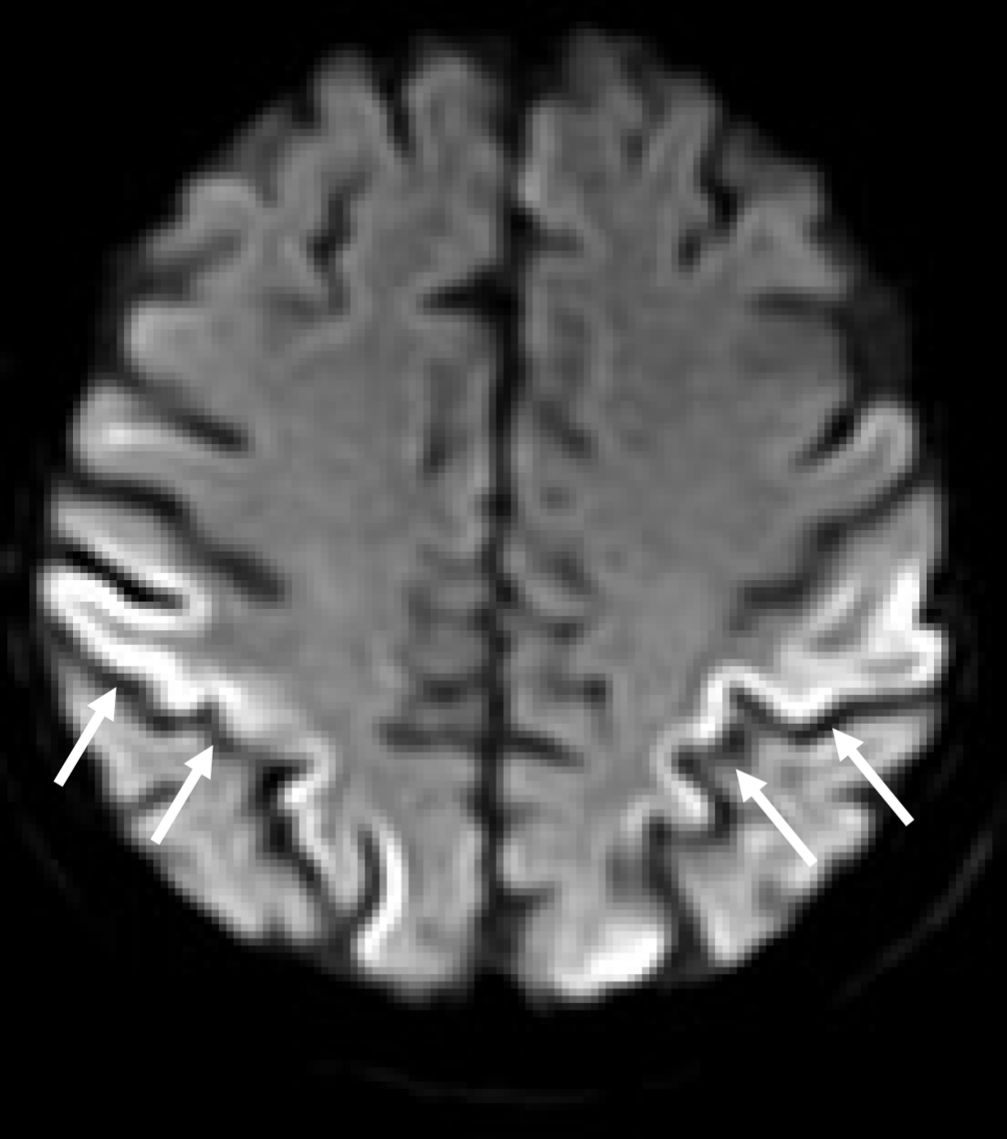
MRI taken upon admission of a 76-year-old woman with pronounced dressing apraxia. Diffusion-weighted imaging showing hyperintense, ribbon-shaped signal irregularities predominately in the parietal cortex bilaterally (white arrows).

Her first cranial magnetic resonance images (MRI) showed marked cortical restriction of diffusion bilaterally (positive “ribbon sign”), most prominent in the parietal regions (Figure 1). A cerebrospinal fluid (CSF) analysis revealed a normal cell count (2/µl; normal <4) and slightly elevated protein (523 mg/l; normal <450). Tests for Borrelia burgdorferi, varicella zoster virus and herpes simplex virus were negative. Her beta-amyloid 1–42 was slightly diminished (544.8 pg/ml; normal >630), and the tau-protein (>1397 pg/ml; normal <290) and phospho-tau-protein (98.5 pg/ml; normal <61) were elevated. Her protein 14-3-3 tested positive, and the first test on PrSc was negative. An EEG at this time showed paroxysmal dysrhythmia with short generalized groups of higher voltage sharp and slow waves. The beginning of CJD was suspected and supportive home care arranged. Seven weeks later, the dressing apraxia had progressed to the point that she needed complete support in dressing. In addition, her dysarthria had also progressed; otherwise, the patient was still ambulatory. A second MRI showed progressive cortical restriction of diffusion. In a second CSF analysis, the pattern of the proteins beta-amyloid 1–42, tau- and phospho-tau-protein were nearly unchanged abnormal. In addition to the positive protein 14-3-3, now PrSc test was positive. After a consultation with the Prion Research Group of University Hospital Göttingen, Germany, probable CJD was diagnosed. As her clinical condition rapidly deteriorated with now more generalized apraxia, inability to walk and appearance of spontaneous myocloni, palliative hospital care was initiated. The patient deceased one week later. A post-mortem examination was not performed.

## Discussion

With this case report, we wish to complement the observations on neuropsychological disorders in CJD for three reasons. First, dressing apraxia as an initial and for a certain time exclusive symptom of CJD has sparsely been reported [4]. Most reports of dressing apraxia are due to cerebrovascular disease, tumors and slowly progressive neurodegenerative diseases [5]. However, in general, neuropsychological symptoms in sporadic CJD are frequent in the early phase of the disease, whereby amnesia, impaired attention, frontal lobe syndrome, aphasia and apraxia in a wide sense are the most frequent [1]. Based on the findings of our patient, dressing apraxia should be added to these neuropsychological observations. Second, the pronounced “ribbon sign” in a diffusion-weighted MRI led to the suspicion of CJD. This radiological manifestation resembles the phenomenon of the Heidenhain variant with predominant cortical affection of the occipital lobe [6]. It is deduced that the clinical symptomatology corresponds to the affected cortical region: The involvement of posterior brain regions may lead to visual disturbances (Heidenhain variant), posterior cortical dementia or even Balint syndrome [4,6,7]. In our patient the parietal lobe was apparently affected as demonstrated in diffusion weighted MRI. Lesions of the parietal lobe are known to be related with apraxia, pantomimic disorders and particularly with dressing apraxia [5,8,9]. Third, a number of radiological CJD mimics of CJD have been recognized including immune-mediated encephalitis, infections, toxic metabolic syndromes, stroke or common neurodegenerative disorders such as Alzheimer disease [10,11]. However, clinical presentation mimics should be borne in mind, too. In our patient, for example, clinically an early corticobasal degeneration would have been a potential differential diagnosis after the first clinical contact [12]. In conclusion, the differential diagnosis of dressing apraxia should be expanded to include early CJD. In the presented patient, in addition to CSF analysis, MRI with diffusion-weighted imaging and course of disease contributed to the diagnosis.
